# Effect of Zinc supplementation on child development: a systematic review and metaanalysis Protocol

**DOI:** 10.22037/ijcn.v15i1.22515

**Published:** 2021

**Authors:** Soheila SHAHSHAHANI, Firoozeh SAJEDI, Shiva FATOLLAHIERAD

**Affiliations:** 1 Pediatric Neurorehabilitation Research Center, University of Social Welfare and Rehabilitation Sciences, Tehran, Iran.

**Keywords:** Child, Child Development, Zinc, Dietary Supplements

## Abstract

Child development is one of the principal aspects of pediatrics. It is a multidimensional process, on which many factors may have different effects. Zinc is a nutritional trace element that has an essential role in neuronal activity and, consequently, in brain development. Since Zinc deficiency is prevalent in developing countries, some clinical trials were conducted to evaluate the impact of zinc supplementation on child development. Thus, we decided to run a systematic review in this area to identify the effectiveness of zinc supplements on child development.

This systematic review protocol will include randomized controlled trials studies (RCTs) in which zinc supplementation was used versus placebo or no intervention, zinc supplementation with other micronutrients versus the same micronutrients without zinc. We will evaluate the effect of zinc alone and zinc co-supplementation with iron on child development. We will search the Medline, Pubmed, EMBASE, ERIC, Psychinfo, the Cochrane Central Register of Controlled Trials (CENTRAL), clinicaltrials.gov, WHO International Clinical Trials Registry Platform (ICTRP), ISRCTN Registry CINAHL, Web of Science and Scopus databases. The clinicaltrials.gov and the Cochrane Library website will also be searched for randomized trials which were registered and completed but not published yet. Two researchers will independently screen titles and abstracts of citations and read the full texts of potentially relevant studies. The data extraction and quality assessment of the papers will be done independently. Any disagreements that arise between the reviewers in the above-mentioned steps will be resolved through discussion. We will report our findings based on the Preferred Reporting Items for Systematic Reviews and Meta-Analyses (PRISMA) guidelines and use the Cochrane Collaboration’s tool for assessing the risk of bias. We will aim to synthesize the results in a meta-analysis if the interventions are similar in methods. Based on the similarities and differences of primary studies, we will use the best statistical methods.

This is a protocol of systematic Review and meta-analysis of the effect of zinc supplementation on child development.

The strengths of this protocol after meta-analysis are as follows**:**

We will identify the strengths and weaknesses of each study.

We will also study if zinc alone and zinc co-supplementation with iron are useful for improving child development in terms of their age, their nutritional status, dose of the zinc supplementation, type of the zinc supplementation (salt), duration of the intervention and iron or other nutrient co supplementations.

We will assume that the measures used for the outcome will be heterogeneous between studies. We know that each study has its own quantity. We will use the random effect models for these heterogeneous data.

## Introduction

Child development is one of the most important aspects of pediatrics. It ensures that children who grow up in the right direction can provide an opportunity for a healthy life. Therefore, the health promotion of children is one of the most important tasks, for which governments must be accountable. In recent years, many studies have been conducted on child development, which have identified some factors that may lead to developmental promotion or disorders of children, as well as finding ways that can prevent their developmental delays. Many studies showed that 10-16% of children worldwide have developmental disorders (1, 2). To put it differently, more than 200 million children under five cannot reach their potential developmental abilities(3). 

Child development is a multidimensional process, which can affect different factors. One of the important and impressive factors is nutrition. Zinc, which is an essential nutritional trace element in human health and wellbeing, neuronal activity as well as brain development (4). Many studies have been investigated on cell maturation, cell division, and brain development (5-7).

 Zinc deficiency is a health problem in undeveloped or developing countries. It is a prevalent issue among the undernourished children and may result in their poor development (8). An estimated 17.3% of the world’s population is at risk of inadequate zinc intake (9). The main intervention strategies for the prevention of zinc deficiency include dietary modification, supplementation, and fortification. Therefore, many health policymakers in underdeveloped regions have decided to use zinc supplementation in the primary health care services. 

Some researchers evaluated the effect of zinc supplementation on child development. In some trials, zinc was prescribed for young children. One study on Canadian infants showed that zinc supplementation improved the motor development of low birth weight (LBW) in infants (10, 11). Friel et.al gave zinc and copper to very-low-birth-weight infants and improved their motor development (10). Black et al. examined the effect of iron, zinc, iron plus zinc, and a micronutrient mixture (MM) containing iron and zinc on 221 rural Bangladeshi infants. They found that zinc had a beneficial effect on infant motor development when proscribed together with iron or as MM (12). In another study on 150 term Chilean neonates of low socioeconomic status who received supplemental zinc, a beneficial effect was found on the mental development and motor quality behavior of healthy term infants (13). When the micronutrient supplementation was given to urban and rural children in china along with zinc, their range of neuropsychological function was much more improved than when they took micronutrient alone(14) . Studies conducted in Indonesia (15) and Brazil (16) did not show any effect of zinc supplements on infant development. In another study, a zinc did not have any beneficial effect on the motor development of infants at 10 months of age (17). In a similar way, there were no relations between zinc supplementation and motor development of rural Guatemalan infants(18) . Ashworth et al. evaluated the effect of zinc on psychomotor development of term LBW infant. They discovered that zinc supplementation was not associated with psychomotor development index (PDI) or mental developmental index (MDI) improvements (16). Taneja et al. evaluated the effect of zinc supplementation on mental and psychomotor scores in children aged 6 to 30 months. They concluded that Zinc supplementation did not affect the mental or psychomotor development index scores (19).

In this area, there have been some systematic reviews and meta-analyses. For instance, a meta-analysis in 2013 by Nissensohn evaluated the effect of zinc on mental and motor development in infants. Their findings showed that zinc supplementation did not seem to affect the mental or psychomotor neurodevelopment of infants. They only included those RCTs that used Bayley as an assessment tool (20). Brown et al. (2009) measured the impact of preventive zinc supplementation on morbidity, mortality, physical growth, and the indicators of behavioral development. According to their findings, zinc supplementation had no significant effects on children's behavioral development (21). Di-ling et al. showed that zinc supplementation had no effects on psychomotor and mental development in infants(22). Gogia and Sachdev assessed the effect of zinc on the mental and motor development of children under 5, showing that there was no significant effect of zinc supplementation (23). 

Other trials did not find any differences or negative effects in the use of zinc supplementation for the motor performance of infants (4, 6, 15, 24) . Since the results of zinc supplementation in the development of the child showed contradictory findings, we decided to conduct a regular study to determine the effectiveness of zinc and zinc supplementation with iron in the development of the child. The main difference between our SR and the previous studies is that they only assessed the motor or mental development of infants or children under five years of age. We decided to assess the gross and fine motor in children under five as well as evaluate the cognition and adaptive behavior domains in children under 42 months (those that evaluated by in any of the first, second, or third edition of Bayley). We will assess whether the effects of zinc, iron, and other nutrient supplements on child development differ from zinc supplementation alone and whether or not the children's nutritional status alters the effects of zinc on growth. On the other hand, since the last SR paper on child development was conducted in 2013, we will also consider the results of RCTs published in recent years.

Objectives 

The primary objective of this systematic review is to assess the effect of zinc alone and zinc co-supplementation with iron on child development in gross motor and fine motor domains of development. Therefore, we will include any articles that evaluated the effect of zinc supplementation in children up to 5. 

The secondary objectives are to identify potential sources of heterogeneity in primary studies and detect the potential differences in the effectiveness of zinc supplementation associated with cognition and adaptive behavior domains in children less than 42 months. Thus, we will only include those trials that used Bayley as an evaluating tool.

## METHODS & ANALYSES

**Eligibility criteria**

Study characteristics

This systematic review will include randomized controlled trials studies (RCTs), open-label RCTs, single-blind RCTs, triple/double-blind RCTs and cross-over RCTs in which zinc supplementation was used versus placebo or no intervention, zinc supplementation with other micronutrients versus the same micronutrients without zinc and the authors evaluated the effect of zinc alone and zinc co-supplementation with iron on child development. The exclusion criteria will be the zinc supplementation as a part of multiple micronutrient interventions.

Types of participants: In this systematic review, we will target those studies with children up to 5 who received zinc supplementation. The exclusion criteria will be children with HIV, developmental dela-y, or developmental disorders, such as autism, Attention-Deficit/Hyperactivity Disorder (ADHD), Intellectual disability (ID). 

Setting and time frame:

No limitation will be considered in this systematic review for setting and time frame.

Report characteristics:

In this systematic review, we selected only those articles with English abstracts and those published at the time of the search. 

Information sources:

We will search PubMed, MEDLINE (Ovid), EMBASE (Ovid), Cochrane Central Register of Controlled Trials (CENTRAL), PsycINFO (Ovid), ERIC (Ovid), CINAHL (EBSCO), Web of Science and Scopus. References of included studies and previous related review articles will be screened to identify other possible relevant studies. Clinical trials registers clinicaltrials.gov, WHO International Clinical Trials Registry Platform (ICTRP), and ISRCTN Registry will also be screened. Furthermore, The American Journal of Clinical Nutrition will be hand searched for other probable relevant articles. To search the databases, we will use Medical Subject Headings (MeSH terms) and text words. As the first step, we will develop and complete the search strategy in Ovid MEDLINE, and then we will adapt the same strategy to search the other databases. There is no language restriction in the search strategy, but we only include the published studies with English abstracts. We use Google translation to translate the non- English retrieved studies into English. This systematic review will be under the Preferred Reporting Items for Systematic Reviews and Meta-Analyses (PRISMA) statement.

**Search strategy**

 Our initial search syntax for MEDLINE will be:

1: zinc.tw.

2: Zinc/

3: 1 or 2

4: Infant, Newborn/

5: Infant/

6: Child, Preschool/

7: Child/

8: (neonate* or infant* or newborn* or baby or babies or toddler* or boy* or girl* or child* or preschool* or pre-school* or school* or schoolchild* or preteen* or pre-teen* or preadolescen* or pre-adolescen*).tw.

9: 4 or 5 or 6 or 7 or 8

10: randomized controlled trial.pt.

11: randomized controlled trial.ab.

12: controlled clinical trial.pt.

13: controlled clinical trial.ab.

14: randomized.ab.

15: randomly.ab.

16: placebo.ab.

17: trial.ab.

18: groups.ab.

19: drug therapy.fs.

20: 10 or 11 or 12 or 13 or 14 or 15 or 16 or 17 or 18 or 19

21: 3 and 9 and 20

**Study records**

*Selection process:*

We will import all search citations into Endnote and remove the duplication. The two referees will then evaluate the citations independently according to the criteria in the titles and abstracts. They will categorize the articles into three groups as relevant, unsure, and not relevant. Those articles, classified as not related by both reviewers, will be excluded from the study. We will resolve any disagreements via consensus discussions. Then, two review authors read the full-texts of relevant surveys and extract their data independently. They will also make a list of articles to be included and code the quality of the papers, independently. Any disagreements will be resolved via consensus discussions by the research team, who will make the final decision.

*Data management:  *For each included study, we will extract the data using an extraction sheet and record them via a piloted data collection form. We will discuss any disagreements among the extracted data to reach a consensus. When necessary, we will contact the authors of the papers for clarification, further information, or the supply of missing data. 

We will report the characteristics of included studies in the "table of Characteristics of included studies." The included data items in the data extraction forms are the author(s), year of publication, study design, study location and setting, intervention date, sample size, participants' characteristics, comorbidities, inclusion, and exclusion criteria, methods of intervention, a dose of zinc, frequency of zinc supplementation, type of zinc compound, duration of the intervention, co-interventions, methods, or tools to assess child development, results and method of allocation, blinding of participants and outcome assessors, exclusion of participants after randomization and proportion of losses at follow-up, study outcomes and the key conclusions of the papers. One of the authors will enter the data into Review Manager Software (RevMan 5.3), and another one will check the accuracy of the entered data. 

In this article, we will include a list of excluded studies with the reasons, and we will document all decisions.

**Assessment of risk of bias in included studies**

Two reviewers will separately assess the quality of included studies using the Cochrane Collaboration’s risk of bias tool (25). The risk of biases in each domain (e.g., selection bias, performance bias, attrition bias, detection bias and reporting bias) will be organized as low, high, or unclear risk.

**Data synthesis**

PRISMA flow chart will be used to report the steps in the search strategy. To evaluate the effect of zinc alone and zinc co-supplementation with iron on child development, we will plan to synthesize results in a meta-analysis if the interventions are similar in methods and conduct subgroup meta-analyses. If the standard deviation is not reported in the reviewed articles, we will calculate the missing SDs with the existing information in the article. Continuous data will be analyzed by computing the standardized mean difference with 95% confidence intervals (CIs). For dichotomous data, we will calculate the effect size as Odds Ratios (ORs) with their 95% CIs. Where studies have more than one intervention group (multi-arm studies), we consider groups that use zinc supplementation (as opposed to zinc supplementation) to compare a single pair and in situations where only one intervention arm is relevant to our study. Based on the similarities and differences of primary studies, we will use the best method of fixed-effect or random-effects model to assess the impact of statistical heterogeneity. After categorizing the studies and performing the data synthesis, the final report will be prepared following the PRISMA guidelines. 

If the quantitative synthesis is not appropriate, we will not perform a meta-analysis, and data will be presented descriptively.

We will use RevMan 5.3 for the risk of bias and STATA 14 for statistical analysis.

**Subgroup analysis and investigation of heterogeneity**

Depending on the sample size of studies and heterogeneity of study populations, we plan to undertake subgroup analyses as follows: age of the children, low birth weight children dose of the zinc supplementation, type of the zinc supplementation (salt), duration of the intervention and other nutrient co supplementations. To limit the risk of multiple comparisons, we will conduct subgroup analyses only on the primary outcomes.

**Sensitivity analysis**

In this review, we will conduct sensitivity analyses to determine whether or not the findings are sensitive to restricting the analyses to those studies that judged to be at low risk of bias. Additionally, we will examine if studies with high rates of inadequate blinding or loss to follow-up are more likely to show positive outcomes. We will also undertake the sensitivity analyses to assess the potential impact of missing outcome data. The results of sensitivity analyses will be reported in a summary table.

**Assessment of heterogeneity**

We will assess the statistical heterogeneity by inspecting the forest plots, calculating I^2 ^statistic with 95% CIs and employing a Chi^2^ test of heterogeneity to determine the strength of evidence that heterogeneity is actual.

**Metabias**In this systematic review, funnel plots will be used to assess publication bias. We will follow the recommendations on testing for funnel plot asymmetry as it is described in the Cochrane Handbook for Systematic Reviews for Interventions (25).

**Confidence in cumulative evidence**

The Grades of Recommendation and Assessment and Development and Evaluation (GRADE) approach will be used to assess the quality of evidence (26). 

## Discussion

Meta-analysis of RCTs across dietary supplementations in children is an important step in child development. This study will provide information about the effect of zinc alone or co-supplemented with iron on child development. The results of this systematic review can help the researchers who wish to design new primary or secondary studies concerning this issue by demonstrating the gaps in knowledge. In addition, it can play an important role in improving the internal credibility of future evidence. By abstracting the results and analyzing the conclusions, decision-making can be improved for health policymakers. On the other hand, if the results show the ineffectiveness of zinc, it can prevent wasting resources.

## In Conclusion,

 In this study, we will identify each study's strengths and weaknesses. We hope to find the most useful zinc supplementation methods to translate them into clinical practices. We will disseminate this protocol in a related peer-reviewed journal. 
